# The characteristics of people who inject drugs in the United Kingdom: changes in age, duration, and incidence of injecting, 1980–2019, using evidence from repeated cross‐sectional surveys

**DOI:** 10.1111/add.15911

**Published:** 2022-05-12

**Authors:** Dan Lewer, Sara Croxford, Monica Desai, Eva Emanuel, Vivian D. Hope, Andrew McAuley, Emily Phipps, Emily J. Tweed

**Affiliations:** ^1^ Public Health and Clinical Directorate UK Health Security Agency London UK; ^2^ Department of Epidemiology and Public Health University College London London UK; ^3^ Clinical and Protecting Health Directorate Public Health Scotland, UK Glasgow UK; ^4^ Public Health Institute Liverpool John Moores University Liverpool UK; ^5^ School of Health and Life Sciences Glasgow Caledonian University Glasgow UK; ^6^ MRC/CSO Social and Public Health Sciences Unit University of Glasgow Glasgow UK

**Keywords:** Ageing, demography, epidemiology, heroin, injecting, modelling, United Kingdom

## Abstract

**Background and aims:**

Mortality and drug treatment data suggest that the median age of people who inject drugs is increasing. We aimed to describe changes in the characteristics of people injecting drugs in the United Kingdom (UK).

**Design:**

Repeat cross‐sectional surveys and modelling.

**Setting:**

Low‐threshold services in the United Kingdom such as needle and syringe programmes.

**Participants:**

A total of 79 900 people who recently injected psychoactive drugs in the United Kingdom, recruited as part of the Unlinked Anonymous Monitoring Survey (England, Wales, Northern Ireland, 1990–2019) and Needle Exchange Surveillance Initiative (Scotland, 2008–2019).

**Measurements:**

Age of people currently injecting, age at first injection, duration of injecting (each 1990–2019) and estimates of new people who started injecting (1980–2019).

**Findings:**

In England, Wales and Northern Ireland between 1990 and 2019, the median age of people injecting increased from 27 (interquartile range [IQR], 24–31) to 40 (IQR, 34–46); median age at first injection increased from 22 (IQR, 19–25) to 33 (IQR, 28–39); and median years of injecting increased from 7 (IQR, 3–11) to 18 (IQR, 9–23). Values in Scotland and England were similar after 2008. The estimated number that started injecting annually in England increased from 5470 (95% prediction interval [PrI] 3120‐6940) in 1980 to a peak of 10 270 (95% PrI, 8980‐12 780) in 1998, and then decreased to 2420 (95% PrI, 1320‐5580) in 2019. The number in Scotland followed a similar pattern, increasing from 1220 (95% PrI, 740–2430) in 1980 to a peak of 3080 (95% PrI, 2160–3350) in 1998, then decreased to a 270 (95% PrI, 130–600) in 2018. The timing of the peak differed between regions, with earlier peaks in London and the North West of England.

**Conclusions:**

In the United Kingdom, large cohorts started injecting psychoactive drugs in the 1980s and 1990s and many still inject today. Relatively few people started in more recent years. This has led to changes in the population injecting drugs, including an older average age and longer injecting histories.

## INTRODUCTION

The average age of people who inject psychoactive drugs such as heroin, crack cocaine, and methamphetamine has increased in many countries. This is reflected in the age of people who died after using drugs that are often injected. For example, the median age at death among people who died after using opiates in England and Wales was 30 in 1993 and 43 in 2018 [1]. Similarly, the average age of people starting community‐based treatment for opiate dependence increased from 31 in 2005/2006 to 40 in 2019/2020 [2]. This is an international trend, also observed in North America, Australia and other countries [3, 4, 5]. As the population ages, professionals working in drug treatment services have reported changing health needs, with more long‐term conditions and greater need for multidisciplinary support [6].

There are several possible reasons for the increasing age of people who inject drugs. First, there may be a ‘cohort effect’ in which more people started injecting drugs in the 1980s and 1990s than in previous or subsequent periods, with many of these individuals continuing to use drugs for many years. This long duration of drug use is evident from the clients of community drug services today. In England in 2019/2020, 69% of those treated for heroin dependence first used heroin before 2000 [2]. Second, the age at first injection may be increasing, which has been observed for various psychoactive drugs in the United States between 2004 and 2019 [7]. Third, the typical length of time for which people inject may be increasing [8, 9]. This would, *cetera paribus*, mean that the injecting population grows and gets older over time. We are not aware of evidence that would allow us to test this third theory directly, although cohort studies that recruited people who injected drugs in the 1960s and 1970s show long durations of injecting [8, 9] so it may be unlikely that the typical duration of injecting is increasing.

Although the ageing of the population is evident from data on drug‐related deaths and the characteristics of people in treatment, these sources are not specifically representative of people who inject drugs. They are likely biased toward people who have been using drugs for longer or have more severe substance use disorders [10]. They also do not allow investigation of more detailed characteristics of the population including the duration of injecting and trends in incidence of injecting. An understanding of the history of the population may help service planners take a long‐term perspective of the needs of people who inject drugs and anticipate possible changes in the future.

We used data from long‐term recurring cross‐sectional surveys of people who inject drugs in the United Kingdom (UK) to: (i) describe changes in the age of people who inject drugs; (ii) explore ‘cohort’ effects and changes in the age at first injection, which may contribute to population ageing; and (iii) estimate trends in the number of people injecting for the first time.

## METHODS

### Data source

We used anonymised data from two recurring cross‐sectional surveys of people who inject drugs (PWID) in the United Kingdom. First, we used data from the Unlinked Anonymous Monitoring (UAM) survey of PWID, which recruits participants through drug treatment services, needle and syringe programmes, outreach services and other specialist agencies in England, Wales and Northern Ireland [11]. UAM includes a self‐completed questionnaire covering demographics, social factors, health, drug use and injecting practices, and blood or oral fluid samples to test for exposure to blood‐borne viruses. It has run annually since 1990. Second, we used data from the Needle Exchange Surveillance Initiative (NESI), which recruits participants through agencies that provide injecting equipment in Scotland, predominantly community pharmacies [12]. NESI uses an interviewer‐led questionnaire that collects similar data to UAM, and has run every 2 years since 2008. The primary objective of both surveys is to monitor the prevalence of blood‐borne viral infections. Approximately one‐third of participants reported participation in earlier survey rounds, and we included these participants on the assumption that sampling is representative of the population and some repeat participation is expected. We included all participants who completed a survey in 2019 or before. Although we had access to data from surveys conducted in 2020, we did not use these data because sampling and participation differed during the coronavirus disease 2019 (COVID‐19) pandemic [13].

**FIGURE 1:**
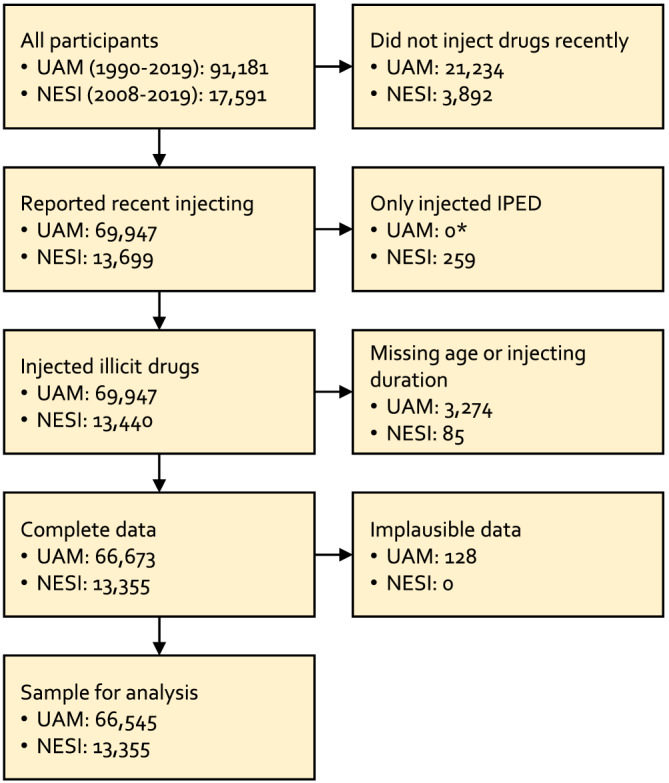
Flow chart of study sample. UAM did not include consistent questions that allowed exclusion of people who inject image and performance‐enhancing drugs, though recruitment is intended to exclude such individuals. UAM, Unlinked Anonymous Monitoring; NESI, Needle Exchange Surveillance Initiative

We excluded: (i) participants who did not inject drugs recently or did not report whether they injected recently. ‘Recent injecting’ was defined as the past 12 months in UAM and 6 months in NESI, because of different availability of consistent questions in each survey. Participants in UAM surveys 1993–1998 were not asked if they had injected in the past 12 months, and we used a question about the calendar year of most recent injection, including participants who reported the same or previous year to the survey completion. UAM surveys 1990–1992 did not include any questions that allowed us to determine recent injecting. However, data from other years show that ~90% of UAM participants in the early 1990s reported recent injecting and we therefore included all participants for 1990–1992; (ii) participants in NESI who reported injecting ‘bodybuilding’ drugs and not psychoactive drugs. UAM did not include consistent questions that allowed exclusion of people who inject image and performance‐enhancing drugs, although recruitment is intended to exclude such individuals and is targeted to people who inject psychoactive drugs; (iii) participants who had insufficient data to derive age, age at first injection, and duration of injection; (iv) participants with implausible data, such as the reported age of first injection being older than the participant's current age. Figure 1 is a flow‐chart showing how the sample was derived.

### Variables

For each participant, we extracted the calendar year and age when the survey was completed, the geographical region, the year of first injection, and age at first injection. In England, geographical regions were defined by nine ‘government offices’, and in Scotland by 14 National Health Services (NHS) Health Boards, which we grouped into three regions (see Table 1). Where participants had missing age, duration of injecting or year of first injection, where possible we calculated these variables based on available data (for example duration of injecting equals current age minus age at first injection).

**TABLE 1:** Characteristics of sample

Variable	Level	England, Wales and Northern Ireland (UAM) No. (%)	Scotland (NESI) No. (%)	*Total* ^a^ *No. (%)*
Total		66 545 (100.0)	13 355 (100.0)	79 900 (100.0)
Sex	Female	16 449 (24.7)	3568 (26.7)	20 017 (25.1)
	Male	49 823 (74.9)	9733 (72.9)	59 556 (74.5)
	Missing/other	273 (0.4)	54 (0.4)	327 (0.4)
Region	East Midlands	4088 (6.1)	–	4088 (5.1)
	East of England	3585 (5.4)	–	3585 (4.5)
	London	10 664 (16.0)	–	10 664 (13.3)
	North East	5512 (8.3)	–	5512 (6.9)
	North West	16 075 (24.2)	–	16 075 (20.1)
	Northern Ireland	1207 (1.8)	–	1207 (1.5)
	South East	6606 (9.9)	–	6606 (8.3)
	South West	9138 (13.7)	–	9138 (11.4)
	Wales	3654 (5.5)	–	3654 (4.6)
	West Midlands	3563 (5.4)	–	3563 (4.5)
	Yorkshire and Humber	2445 (3.7)	–	2445 (3.1)
	Greater Glasgow and Clyde	–	5521 (41.3)	5521 (6.9)
	Lothian and Tayside	–	2698 (20.2)	2698 (3.4)
	Other regions of Scotland	–	5136 (38.5)	5136 (6.4)
	Missing/other	8 (<0.1)	–	8 (<0.1)
Year of survey	1990–1994	11 453 (17.2)	–	11 453 (14.3)
	1995–1999	12 696 (19.1)	–	12 696 (15.9)
	2000–2004	11 351 (17.1)	–	11 351 (14.2)
	2005–2009	11 604 (17.4)	2123 (15.9)	13 727 (17.2)
	2010–2014	10 031 (15.1)	6244 (46.8)	16 275 (20.4)
	2015–2019	9410 (14.1)	4988 (37.3)	14 398 (18.0)

^a^ Total column provided for completeness. Data from UAM and NESI were not combined and all analyses of UAM and NESI data were conducted separately.

Abbreviations: NESI, Needle Exchange Surveillance Initiative; UAM, Unlinked Anonymous Monitoring.

### Analysis

We described the population in four ways:

1. Age of people who inject drugs: we reported the median and interquartile range (IQR) of the age of participants by survey year. We used quantile regression [14] to estimate these values by year. In this model, the dependent variable was the age at survey completion and the independent variables were linear and quadratic terms of the calendar year of survey. Because of our large sample, the confidence intervals for quantile estimates were narrow and we focused on the distribution of participants' age rather than the precision of our estimates, although confidence intervals for quantile estimates are provided in the Supporting information.
2. Age at first injection: we limited the data to participants who started injecting in the past 3 years to minimise bias related to exiting the population, for example because of cessation of injecting or death. Including the full sample may lead to underestimates of the age at first injection, because participants who started injecting many years before completing the survey are likely biased toward those who started at a younger age. This analysis estimates the age of initiation by reported year of initiation, rather than by the year when surveys were completed. The age at which participants first injected in a given year (e.g. 2000) can be derived from surveys conducted in multiple calendar years (e.g. 2000–2019), although we limited analysis to the subsequent 3 years (e.g. 2000–2002) to minimise bias. We used the method in (1) to estimate quantiles of the age at first injection by calendar year of initiation.
3. Duration of injecting: we used the method in (1) to describe the duration of injecting by survey year. We also reported the distribution of year of initiation by calendar year of survey.
4. Number of people injecting for the first time each year: we used the distribution of dates of initiation by survey year to create modelled estimates of the size of new cohorts. For example, if a larger proportion of participants surveyed in 2019 started injecting in the 1990s than in the 2000s, we can infer that more people started injecting in the 1990s than in the 2000s. We assumed that people have a constant probability of stopping injecting, such that there is exponential decay in the number continuing to inject from any given cohort. We chose a mean duration of 15 years following other modelling studies that have assumed mean injecting durations of 20 [15], 8 [16] and 11 [17] years. We used this assumption to infer the relative numbers of people starting. We used a Monte‐Carlo method to combine evidence from all survey years, which is detailed in the Supporting information together with data, analysis code and sensitivity analyses. We used existing estimates of the number of people injecting opiates and crack cocaine in England for 2011 [18] and PWID in Scotland for 2006 [19] to translate the results of this model into estimates of the absolute number of people who first injected drugs each year. We are not aware of estimates of the prevalence of drug injection in Wales, and therefore, did not estimate the number of people injecting for the first time in Wales.

We stratified each analysis by region. We did not report results separately for Northern Ireland because of a small number of participants. Selected region‐specific results are provided in text, with full results in the Supporting information.

The analysis plan was not pre‐registered and results should be considered exploratory.

Analysis was done using R version 4.0.3.

### Approvals

The UAM survey has ethical approval from Public Health England and the London Research Ethics Committee (98/2/051). Ethical approval for the NESI survey was granted by the National Health Service West of Scotland Research Ethics Committee (reference 08/S0709/46). These approvals apply to analyses of anonymised data and data were fully anonymised before analysis.

## RESULTS

The study included 79 900 participations by people who had recently injected psychoactive drugs. 66 545 (83%) were from UAM and 13 355 (17%) from NESI. Characteristics are summarised in Table 1. Across both surveys, 74% (59 556/79 990) were male; the largest numbers in UAM were from the North West, London and the South West of England, from Greater Glasgow and Clyde in NESI. Most had injected heroin. In UAM, participants reported which drugs they had recently injected in surveys conducted from 2006; 92% (19 989/21 790) reported recent heroin injection. In NESI, all participants reported which drugs they injected, and 96% (12 758/13 355) reported recent heroin injection.

### Age of people who inject drugs

**FIGURE 2:**
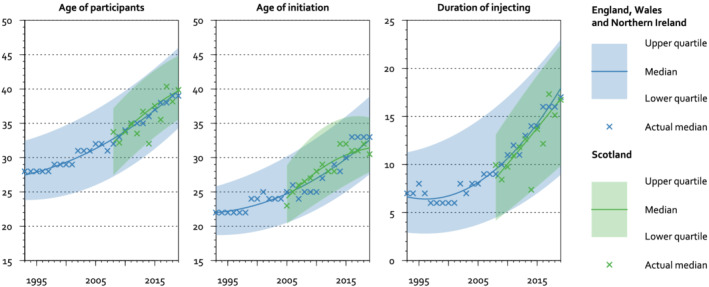
Age of UAM participants, age at first injection of psychoactive drugs, and duration of injecting, by survey year. UAM, Unlinked Anonymous Monitoring

The age of participants who report recently injecting drugs increased over time. In UAM, the median age increased from 27 (IQR 24–31) in 1990, to 33 (IQR 27–39) in 2008, and 40 (IQR 34–46) in 2019. In NESI, the median age increased from 32 (IQR 27–37) in 2008 to 40 (IQR 36–46) in 2019 (Figure 2). The age of participants varied between regions. In 2019, the median age was oldest in London and the North West and youngest in the North East and Lothian and Tayside. In some regions of England, the median age of participants followed a ‘U’ shape over time, decreasing during the 1990s, and then increasing. This U shape was most prominent in the East Midlands, West Midlands, South West and North East. In other regions of England, including London, the South East and the North West, age increased in an approximately linear manner across the whole period. In Scotland, age increased in all regions from 2008 to 2019. Participants in Greater Glasgow and Clyde were older than those in other regions of Scotland (Supporting information).

### Age at first injection of psychoactive drugs

The age at first injection increased over time. In the UAM, the median age increased from 22 (IQR 19–25) in 1990, to 26 (IQR 22–32) in 2008, and 33 (IQR 28–39) in 2019. In NESI, the median age increased from 27 (IQR 22–32) in 2008 to 31 (IQR 28–36) in 2019 (Figure 2). The age at first injection varied between regions. In 2019, people injecting for the first time were oldest in London (median 36) and the West Midlands (median 35) and youngest in the South West (median 30) and ‘other regions of Scotland’ (median 31).

### Duration of injecting

The duration of injecting increased over time. In the UAM, the median duration increased from 7 years (IQR 3–11) in 1990, to 9 years (IQR 4–16) in 2008, and 18 years (IQR 9–23) in 2019. In NESI, the median duration increased from 9 years (IQR 4–13) in 2008 to 17 years (IQR 10–22) in 2019 (Figure 2). The duration of injecting varied between regions. In 2019, participants had injected for longest in London and Yorkshire and Humber (median 20 years in both regions) and shortest in Lothian and Tayside and East of England (median 15 years in both regions). As with the age of the population, in some regions of England the duration followed a U shape, first decreasing from 1990 until the late 1990s and then increasing. This U shape was most prominent in the same regions as for the age of the population: the East Midlands, West Midlands, South West and North East.

**FIGURE 3:**
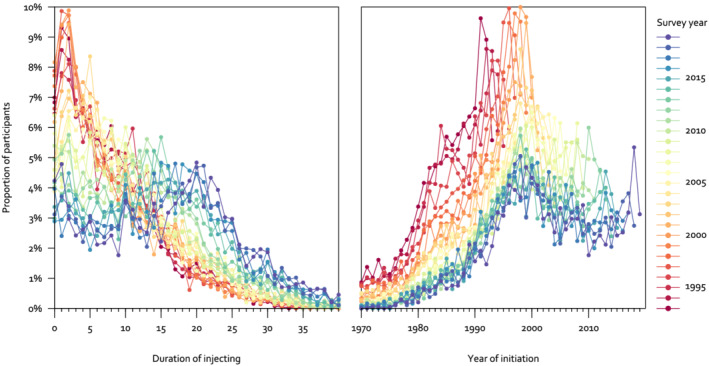
Duration of injecting among people who inject psychoactive drugs in England, Wales, and Northern Ireland, 1993–2019. *Note*: A similar figure for Scotland using data from NESI is provided in the Supporting Information. The pattern is similar with a peak of initiation in the mid‐late 1990s. NESI, Needle Exchange Surveillance Initiative

The distribution of duration changed over time. In the early 1990s the distribution in UAM was right‐skewed with modal durations of 1 or 2 years, whereas in later years the distribution was wider and in 2018 and 2019 the modal duration was 20 years (Figure 3, left panel). When we plotted these data by year of initiation rather than duration of injecting, there was a clear peak of initiation in the mid‐late 1990s (Figure 3, right panel). The modal years of initiation for participants in 2017, 2018 and 2019 were 2000, 1998 and 1999, respectively.

### Estimates of the number of people injecting for the first time each year

**FIGURE 4:**
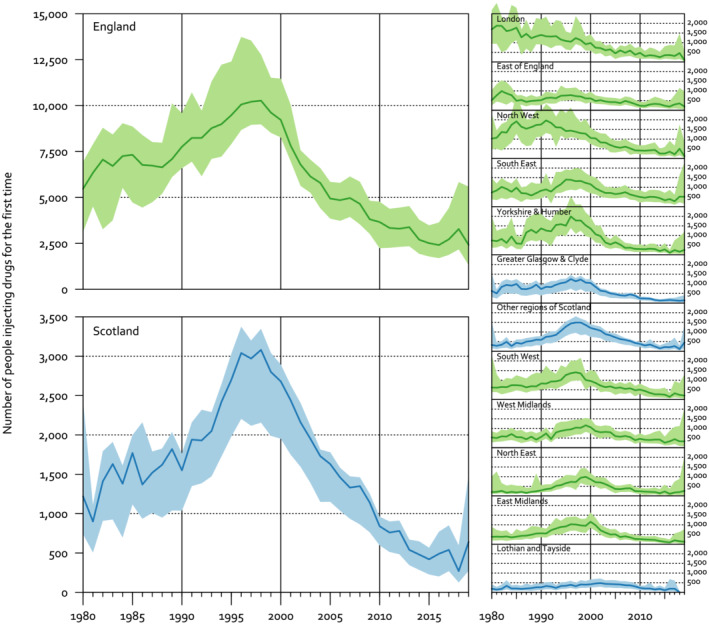
Modelled number of people injecting psychoactive drugs for the first time each year, with 95% prediction intervals

We estimated that the number of people who started injecting psychoactive drugs annually in England increased from 5470 (95% prediction interval [PrI], 3120–6940) in 1980 to a peak of 10 270 (95% PrI, 8980–12 780) in 1998, and then decreased to 2420 (95% PrI, 1320–5580) in 2019 (Figure 4). The estimates for the final years are imprecise because they are based on a small number of surveys. The number of people injecting for the first time in Scotland followed a similar pattern, increasing from 1220 (95% PrI, 740–2430) in 1980 to a peak of 3080 (95% PrI, 2160–3350) in 1998, then decreased to a 270 (95% PrI, 130–600) in 2018.

The timing of the peak differed between regions, with an earlier peak in London and the North West of England. For example, in 1981 we estimated that 1880 (95% PrI, 950–3050) people injected for the first time in London and 210 (95% PrI, 120–1270) in the North East. In 1999, the North East had overtaken London, with 920 (95% PrI, 590–1320) people injecting for the first time in London and 990 (95% PrI, 570–1440) in the North East. The North West, East of England, South East, Yorkshire and Humber and Greater Glasgow and Clyde also appear to have had earlier or less pronounced peaks than other regions of the United Kingdom.

**FIGURE 5:**
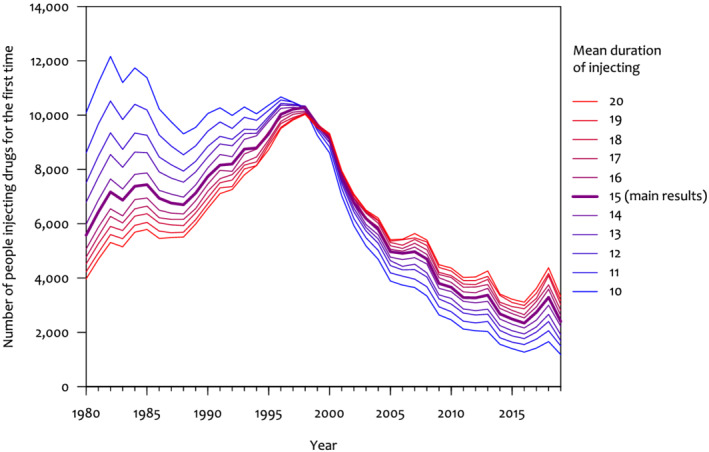
Sensitivity analysis of the effect of varying the assumed typical duration of injecting on the modelled number of people injecting for the first time each year in England (point estimates only)

We did a sensitivity analysis varying the assumption that the mean duration of injecting is 15 years, from 10 years to 20 years. This showed that shorter assumed durations of injecting inflated the estimated number that started injecting in earlier years (e.g. the 1980s) and deflated the number starting in later years (e.g. after 2010). Conversely, longer injecting durations deflated the estimated number that started in earlier years and inflated the number starting in later years (Figure 4,5). In all scenarios, the number of people injecting for the first time reduced rapidly from 1997 onward. We report these sensitivity analyses in more detail in Supporting Information.

## DISCUSSION

### Key findings

We used data from repeated cross‐sectional surveys spanning 29 years to show the changing population structure of PWID in the United Kingdom. In the 1990s, relatively large numbers started injecting, and the population was mostly people in their 20s who had injected for fewer than 10 years. In the years after 2000, the number of people taking up injecting reduced, and by 2019 people who injected drugs were mainly in their 30s and 40s and had injected for 15 or more years. These demographic changes varied across the country with an earlier ‘wave’ in London and the North West of England, where numbers peaked in the 1980s rather than 1990s, and hence, PWID in these regions today are older.

The increasing age of PWID is a different phenomenon to ageing in the general population. Among PWID, the increasing age is largely a ‘cohort effect’, in which the average age is driven by a group of individuals who first injected in the 1990s. The increase in the average age of the general population is slower and is primarily driven by reducing mortality rates and decreasing fertility rather than different sized age cohorts [20].

### Comparison with other studies of time trends in psychoactive drug injection

Crime and drug treatment records suggest that heroin use was rare before the 1980s, and then increased in the 1980s with new supply routes from the Middle East [21]. Local data showed relatively large numbers of people using heroin in the 1980s in some cities such as London, Liverpool, Manchester, Bristol, Edinburgh, Dundee, and Glasgow [22, 23, 24, 25, 26]. A study of the Wirral, an area in the North West of England with a high level of social deprivation in the 1980s, documented a large increase in the number of new users of heroin in the mid‐1980s, with estimates broadly compatible with our modelled estimates for the North West of England [27]. Data from treatment services and surveys of professionals suggested that other parts of the United Kingdom had smaller numbers people using heroin in the 1980s, but experienced a ‘second wave’ in the 1990s, whereas the urban areas where the ‘first wave’ of the 1980s focused did not all experience this second wave [21, 28]. Two possible reason why these areas were less affected by the second wave of the 1990s are (i) these areas had already established harm reduction services and education given their experience in the 1980s and (ii) incidence of drug use can be understood in a similar way to infectious disease outbreaks, in which the number of ‘susceptible’ individuals accumulates until an ‘outbreak’ occurs, and after the outbreak there is a period of ‘herd immunity’ when social networks are saturated and current users know few potential users [21, 29].

A study estimating the number of new users of opiates based on the number of opiate‐related deaths, conducted in 2004, suggested that the national number of new users increased substantially in the 1990s and peaked around 1996 [30]. The absolute numbers in this study were larger than our estimates, which may be because it estimated the number of all opiate users, whereas we focused on people who inject drugs (likely a smaller population), or because of other methodological differences. Data from general practitioners also suggested reducing incidence of new opiate users after 1998 [31]. Taken together, this evidence and our study show a consistent pattern of growing numbers of new users in the 1980s and 1990s and then a decline from the late 1990s and an earlier peak in London and some other urban centres.

‘Waves’ of injecting have been observed internationally since the mid‐20th century. The idea of ‘epidemics’ of heroin use was discussed in the United States in the 1970s, following the observation that new users ‘spiked’ and then quickly decreased within geographical areas. Hunt and Chambers [29] suggested that heroin use spreads primarily through social networks (rather than marketing by drug dealers, for example), which is supported by survey data from the United Kingdom [27]. This ‘micro‐diffusion’ drives local outbreaks, first in larger cities, then towns, and then rural areas. The increasing age of people injecting for the first time in our results supports the idea of micro‐diffusion, as the age of new users appears associated with the age of current users. Hunt and Chambers [29] observed that after a town or city experienced a wave, there was usually a gap of decades before a new susceptible population emerged and there may be another wave. Our results suggest that there has not been a wave of psychoactive drug injection in any part of the United Kingdom since the late 1990s. It is possible that there will be another wave in coming years, although the role of injecting may change.

### Relevance for policy and clinical practice

Our results provide a long‐term perspective of how the population injecting psychoactive drugs has changed over time. We show that the characteristics of people who inject drugs in the United Kingdom are primarily determined by a cohort that started injecting in the 1980s and 1990s, which is why the average age and duration of injecting is increasing. For services that support this population, including needle and syringe programmes and community health services that provide opioid agonist therapy, a key question is how to support this cohort.

Ageing is associated with an increasing importance of long‐term health conditions. As the cohort of the 1980s and 1990s has got older, the main causes of death have shifted from infections and drug overdoses to non‐communicable diseases [32]. The prevalence of long‐term conditions is now very high. For example, recent spirometry surveys in community‐based services that treat heroin dependence have found that approximately one in three clients has chronic obstructive pulmonary disease [33]. Qualitative research has found that people who inject drugs have many barriers to accessing health services, including stigmatising attitudes among staff, ‘diagnostic overshadowing’, in which symptoms are not investigated because they are attributed to drug use, and delayed help‐seeking because of low expectations and normalisation of pain [34, 35]. To cope with the increasing health needs and poor healthcare access among their clients, services supporting this population will need greater integration with local health services. This may mean partnerships in which general practitioners or key specialists such as respiratory physicians provide open‐access clinics at drug treatment and outreach centres. If the population dynamics remain stable, with relatively few people starting injecting drugs and continued ageing in the cohort of the 1980s and 1990s, the importance of long‐term conditions will continue to increase.

It is possible that the population dynamics will not remain stable, and there could be another wave of drug injection. Our results show there were waves in the 1980s and 1990s, and other studies show earlier, albeit smaller, waves in the 1960s and 1970s [21, 29]. The fact that incidence of psychoactive drug injection has reduced since the 1990s does not mean incidence will reduce forever. In the United States, transitions from prescribed to illicit opioids may have driven an increase in injecting in the 2000s [36]. A new wave in the United Kingdom would mean different demands on services. In particular, people who started recently tend to be younger and less engaged with harm reduction services, so public health priorities may shift toward outreach rather than support for complex health and social needs.

### Limitations

Our study has three key limitations. First, there are likely to be selection biases in the data. Because of the illicit nature of psychoactive drug use, there are no population registers from which representative samples of PWID can be drawn. UAM and NESI aim to be as representative of PWID in the United Kingdom as possible by recruiting from low‐threshold services that are accessed by the vast majority of PWID [37, 38] and using research methods such as peer outreach and co‐design of surveys that facilitate participation. Nonetheless, survey participants are likely to differ in some ways to the overall population. We believe the most relevant bias to this study is possible underrepresentation of people who have recently started injecting, because this group may have less contact with services such as needle and syringe programmes. This would mean our estimates of the age of the population, age of initiation, and duration of injecting are higher than the population values. It is also possible that this bias has changed over time. For example, if services that support survey recruitment have become progressively less accessible to younger people, this may partly explain the observed results of increasing age of the population. Second, the model of the number of people injecting for the first time was sensitive to our assumption about the rate of cessation. Our sensitivity analysis showed that even in extreme scenarios (average durations of injecting of 10 or 20 years); the overall shape of the injecting epidemic was similar with initiation peaking in the 1990s and reducing after that, although the absolute estimates varied under these different assumptions. Third, our study does not include people who use psychoactive drugs in different ways, such as smoking. It is possible that the declining incidence of injecting is being replaced by other forms of drug use, and this would not be captured in our study.

## CONCLUSION

In the United Kingdom, there are large cohorts of people who started injecting drugs in the 1980s and 1990s and still inject today. This has led to changes in the population structure, including an older average age and longer histories of injecting. Services supporting this population need resources that reflect the increasing health and social needs associated with ageing and long durations of drug use.

## DECLARATION OF INTERESTS

None.

## AUTHOR CONTRIBUTIONS

**Dan Lewer:** Conceptualization; formal analysis; methodology; software; visualization. **Sara Croxford:** Conceptualization; methodology; supervision. **Monica Desai:** Conceptualization; methodology; supervision. **Eva Emanuel:** Conceptualization; data curation; methodology; supervision. **Vivian Hope:** Conceptualization; methodology; supervision. **Andrew McAuley:** Conceptualization; methodology; supervision. **Emily Phipps:** Conceptualization; methodology; supervision. **Emily Tweed:** Conceptualization; methodology; supervision.

### OPEN RESEARCH BADGES

This article has earned an Open Materials badge for making publicly available the components of the research methodology needed to reproduce the reported procedure and analysis. All materials are available at https://github.com/danlewer/uam_nesi.

## Supporting information




**Data S1** Supporting information
