# Supplementary material for: The characteristics of people who inject drugs in the United Kingdom: changes in age, duration, and incidence of injecting, 1980–2019, using evidence from repeated cross‐sectional surveys
Source: Addiction. 2022 May 12;117(9):2471–80. doi: 10.1111/add.15911 (PMC9544601; doi:10.1111/add.15911)
Supplement: Supplementary file 1 — Data S1 Supporting information [file ADD-117-2471-s001.pdf]

Supplementary information

1. Age of participants by region 2
2. Age of initiation by region 3
3. Duration of injecting by region 4
4. Proportion of participants in UAM who report injecting in the past 12 months 5
5. Detailed method of modelling the number of people who injected for the first time each year .. 6
6. Estimated number of people injecting drugs for the first time in England, Scotland, and subregions, 1980-2019 14
7. Histograms of duration of injecting by survey year in Scotland 17
8. References for supplementary information 18

1. Age of participants by region

Figure 1: Age of people who inject drugs in the Unlinked Anonymous Monitoring Survey of People who Inject Drugs (England and Wales) and the Needle Exchange Surveillance Initiative (Scotland)

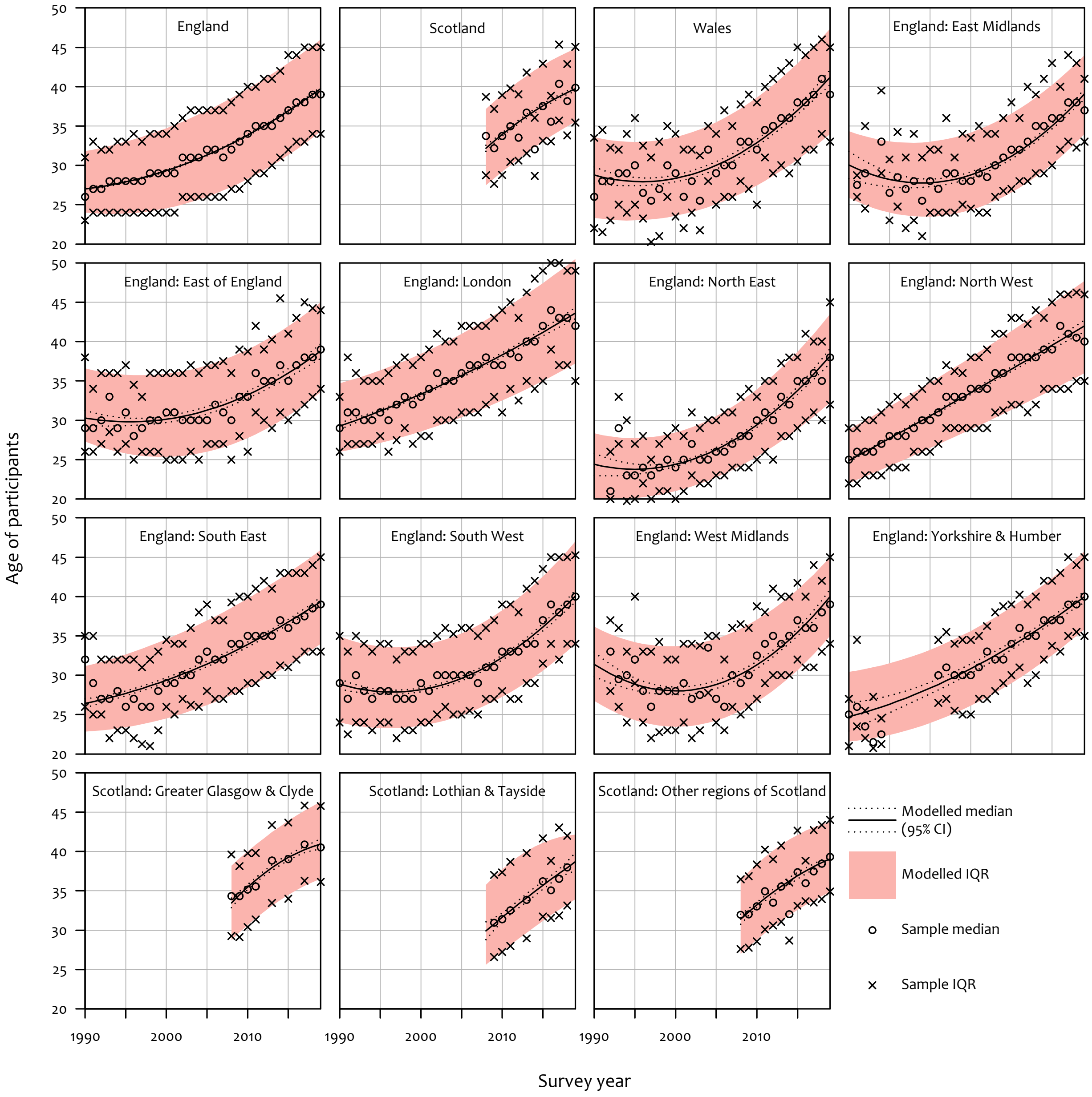

Data and code for this chart is available at https://github.com/danlewer/uam_nesi.

2. Age of initiation by region

Figure 2: Age when people first injected drugs, as reported in the Unlinked Anonymous Monitoring Survey of People who Inject Drugs (England and Wales) and the Needle Exchange Surveillance Initiative (Scotland)

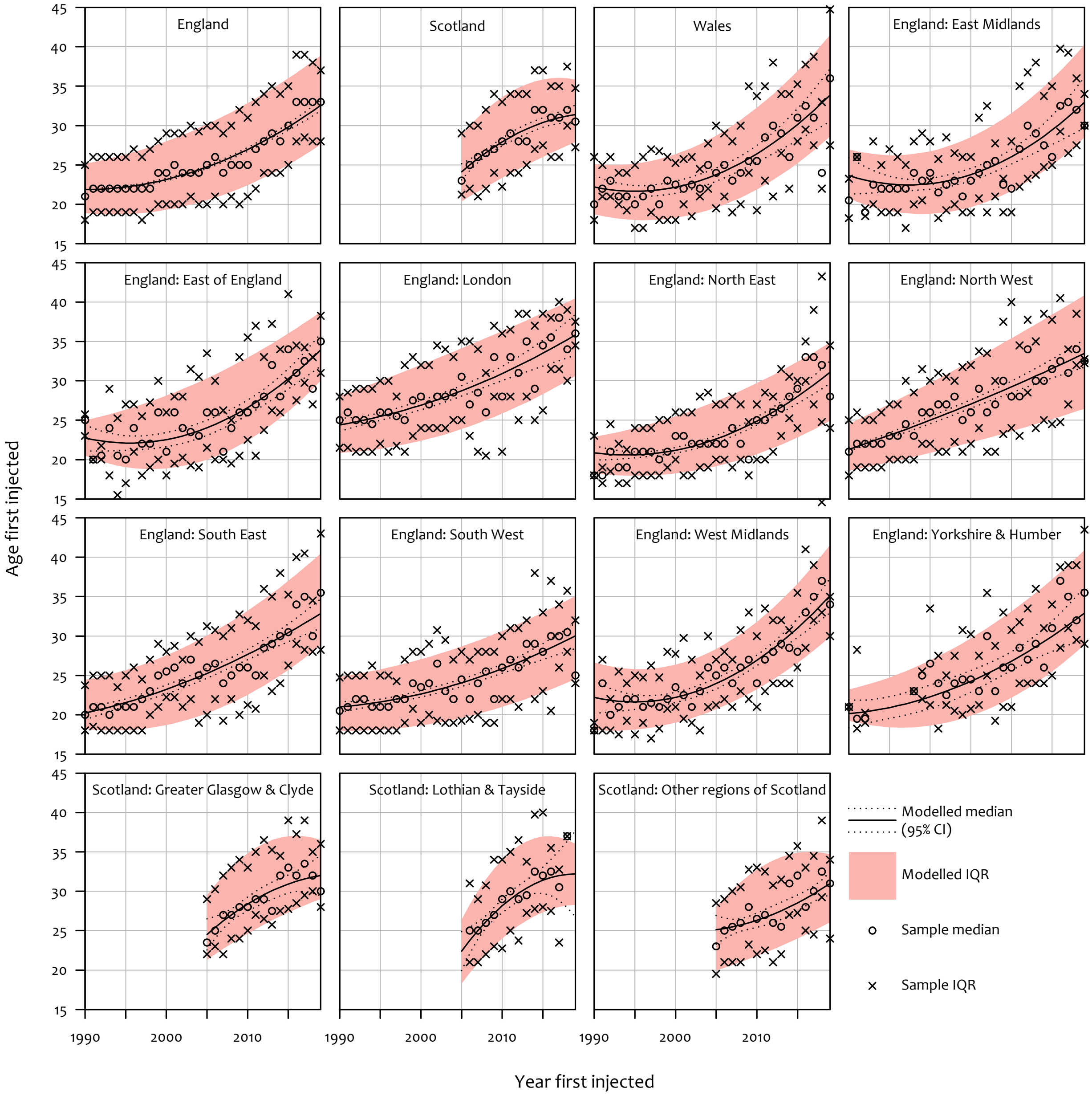

Data presented in this chart is limited to participants who reported first injecting in the three years before participating in the survey. Data and code for this chart is available at https://github.com/danlewer/uam_nesi.

3. Duration of injecting by region

Figure 3: Duration of injecting reported in the Unlinked Anonymous Monitoring Survey of People who Inject Drugs (England and Wales) and the Needle Exchange Surveillance Initiative (Scotland)

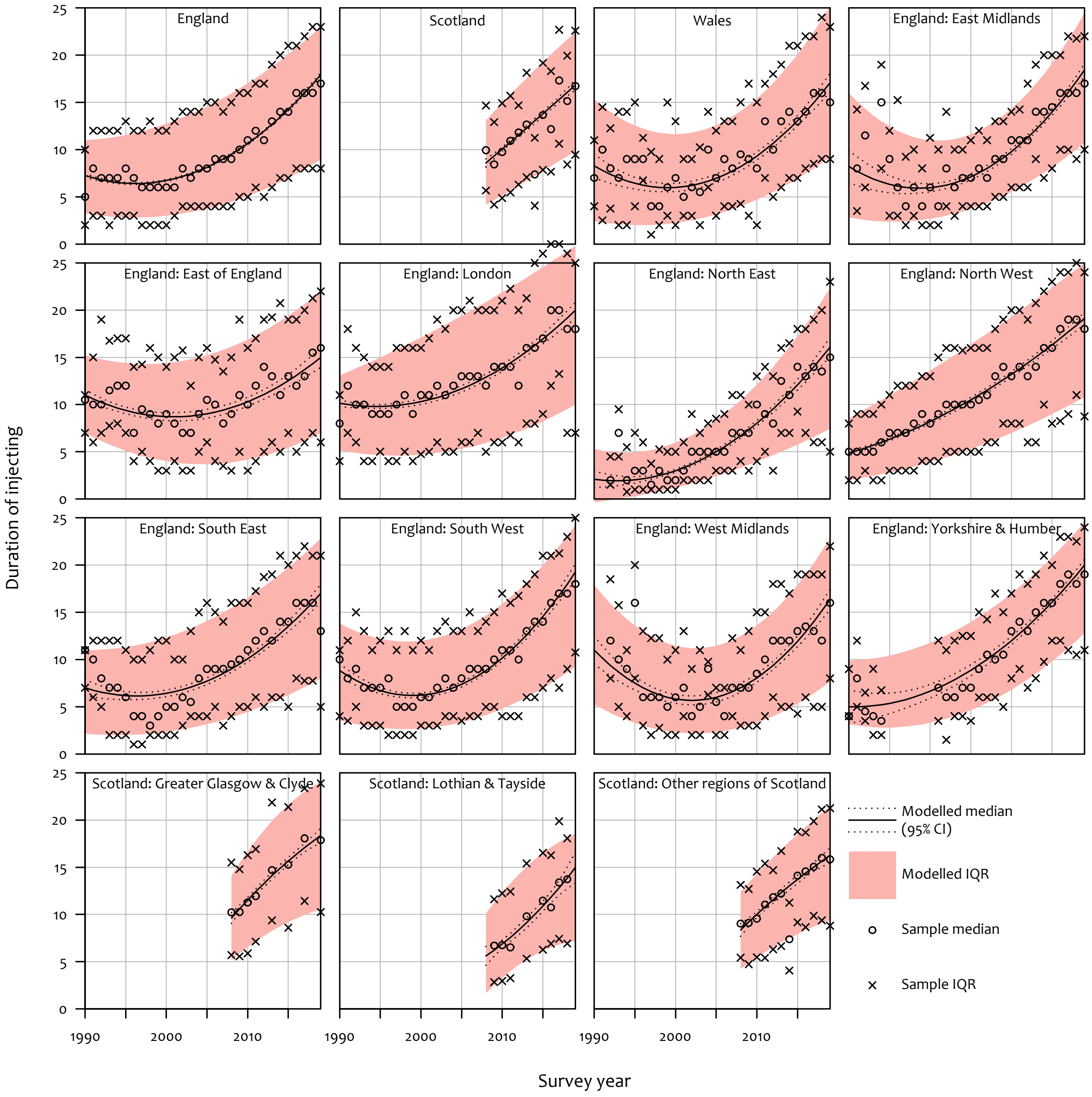

Data and code for this chart is available at https://github.com/danlewer/uam_nesi.

4. Proportion of participants in UAM who report injecting in the past 12 months

Figure 4: Proportion of participants in the Unlinked Anonymous Monitoring Survey of People who Inject Drugs (UAM) that reported injecting in the past 12 months

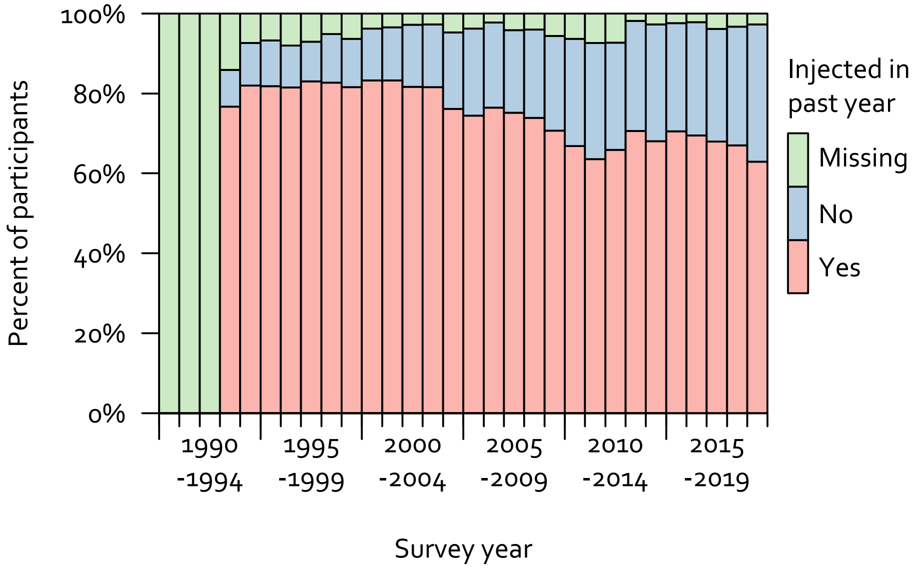

5. Detailed method of modelling the number of people who injected for the first time each year

The UAM and NESI surveys report when they first injected drugs. From this information, we can infer the relative size of new cohorts of PWID. It is clear from figure 3 in the main article, for example, that more people started injected in the late 1990s than in subsequent years.

We used the following method to estimate the size of new cohorts:

(1) **Calculate pairwise ratios of new initiators.** Within each survey year, we calculated the number of participants by year of initiation (limiting to years of initiation from 1980 onwards) and then compared pairs of initiation years. We used an assumption (λ) that the mean duration of injecting was 15 years to estimate a ratio of new initiators between each pair of years. For example, in Table 1, 80 participants reported first injecting in 1980 and 90 reported first injecting in 1983. If we assume that 81.9% (calculated as $e^{(-3/\lambda)}$) of people remain injecting three years after they first inject, we can estimate that the number of new initiators in 1983 was 0.92 times that in 1980.

Table 1: Example of estimation of pairwise ratios of new initiators

Survey year	*Year 1*	*Year 2*	*Difference in time*	*Proportion of new initiators from year 1 remaining in year 2*	*Number starting injecting year 1*	*Number starting injecting year 2*	*Adjusted number in year 1*	*Ratio*
sy	y1	y2	d = y2-y1	$p = e^{(-d/\lambda)}$	N1	N2	aN1 = N1 / p	R = N2/aN1
1990	1980	1981	1	0.94	50	60	53.4	1.12
1990	1980	1982	2	0.88	70	85	80.0	1.06
1990	1980	1983	3	0.82	80	90	97.7	0.92

(2) **Use a bootstrap method to estimate the variance of pairwise ratios.** Resample each survey year 1000 times with replacement and recalculate each ratio, then calculate the variance (σ^2) of the log of each ratio.

Table 2: Example of ratios with variance estimates

sy	*y1*	*y2*	*R*	*log(R)*	*σ^2 log(R)*
1990	1980	1981	1.12	0.05	0.01
1990	1980	1982	1.06	0.02	0.02
1990	1980	1983	0.92	-0.03	0.01

(3) **Combine evidence from different survey years using inverse variance weighting.** An estimate of the ratio of new initiators between each pair of years is available from all survey years y2 and

after. For example, the difference between 2014 and 2017 is available for survey years 2017-2019. We combined these ratios using inverse variance weighting, such that each unique pair of years had a log ratio and a variance (i.e. fixed effects meta-analysis of survey years). Individuals cannot be identified in this data and the full dataset is provided online (https://github.com/danlewer/uam/tree/main/ratios_of_new_initiators), together with analysis code for subsequent steps (https://github.com/danlewer/uam/blob/main/model_new_injectors.R). The heatmap in Figure 5 shows that cohorts of the 1990s were bigger than cohorts of the 1980s, and cohorts of the 2000's were smaller than all earlier cohorts.

Table 3: Ratios of people starting injecting in 2017 vs. 2014 by survey year, with pooled value using inverse variance weighting

sy	y1	y2	log(R)	σ^2 log(R)
2017	2014	2017	0.0006	0.035
2018	2014	2017	0.3136	0.036
2019	2014	2017	-0.1725	0.035
Pooled value	2014	2017	0.0451	0.012

Figure 5: Heatmap of ratios of number of people starting injecting illicit drugs in England, 1980-2019, using the assumption that the mean injecting duration is 15 years, based on pooled ratios from all available survey years

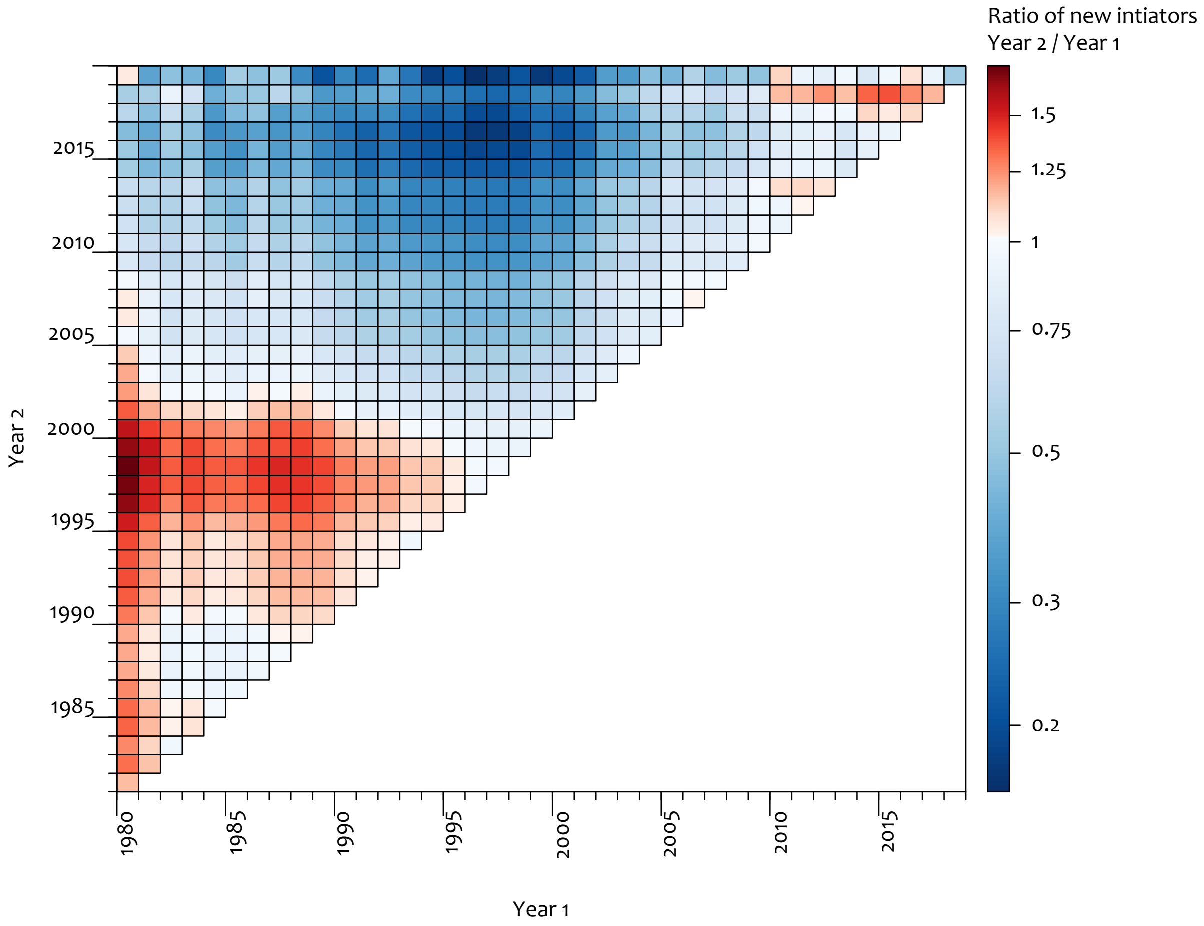

(4) **Use a Monte-Carlo method to generate simulations of the ratio of new initiators vs. 1980.** We generated 1,000 simulations of the ratio of new initiators compared to 1980. For each simulation, we started at a random year between 1980 and 2019. We sampled a new log ratio from a normal distribution with standard deviation of the square root of the variance. We then rescaled the ratios such that 1980 = 1.

Table 4: Example of single simulation where the base year is 1987. Table truncated at 1994 for brevity.

y1	*y2*	*log(R)*	*σ² log(R)*	*σ log(R)*	*Sampled log(R)*	*Sampled R*	*Ratio vs. base year*	*Rescaled to 1980*
1980	1987	0.195	0.002	0.046	0.256	1.292	0.774	1.000
1981	1987	0.053	0.002	0.043	0.063	1.065	0.939	1.213
1982	1987	-0.078	0.002	0.040	-0.100	0.905	1.105	1.428
1983	1987	-0.058	0.002	0.039	-0.044	0.957	1.045	1.350
1984	1987	-0.102	0.001	0.038	-0.076	0.927	1.079	1.393
1985	1987	-0.086	0.001	0.037	-0.060	0.942	1.062	1.372
1986	1987	-0.076	0.001	0.037	-0.069	0.934	1.071	1.384
1987	1987	0	0	0	0	1	1	1.292
1987	1988	-0.002	0.001	0.036	0.091	1.096	1.096	1.416
1987	1989	0.014	0.001	0.035	-0.059	0.943	0.943	1.218
1987	1990	0.106	0.001	0.034	0.111	1.117	1.117	1.444
1987	1991	0.137	0.001	0.034	0.164	1.179	1.179	1.523
1987	1992	0.167	0.001	0.033	0.171	1.186	1.186	1.533
1987	1993	0.186	0.001	0.032	0.242	1.274	1.274	1.646
1987	1994	0.191	0.001	0.034	0.169	1.184	1.184	1.529

(5) **Estimate point estimates and prediction intervals for the ratio of new initiators vs. 1980.** As a point estimate, we took the median simulated ratio of new initiators vs. 1980. As prediction intervals, we took the 0.025 and 0.975 quantiles.

Figure 6: Estimate of the ratio of people starting injecting illicit drugs in England, compared to 1980. Band shows 95% prediction interval

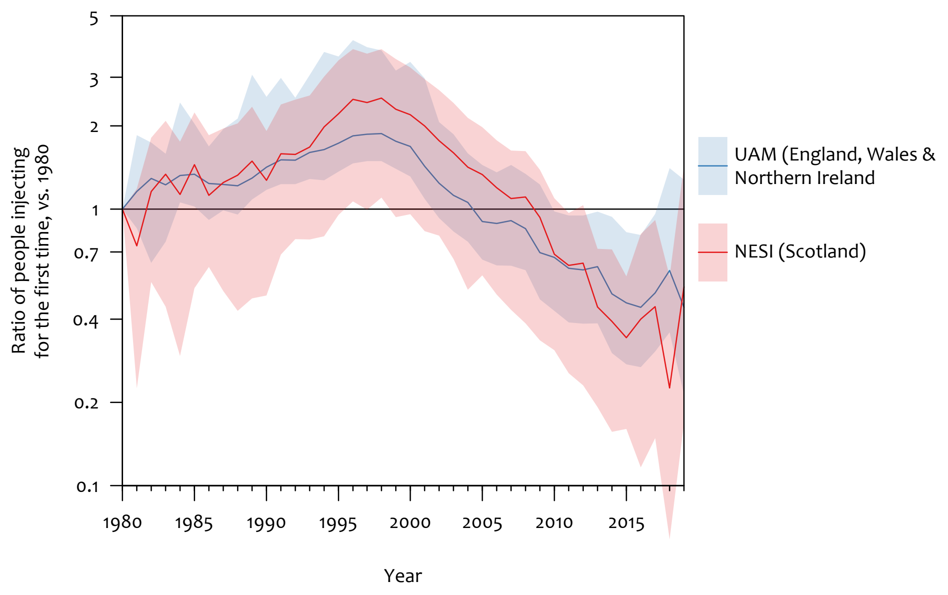

Figure 7: Results of sensitivity analysis varying the average duration of injecting

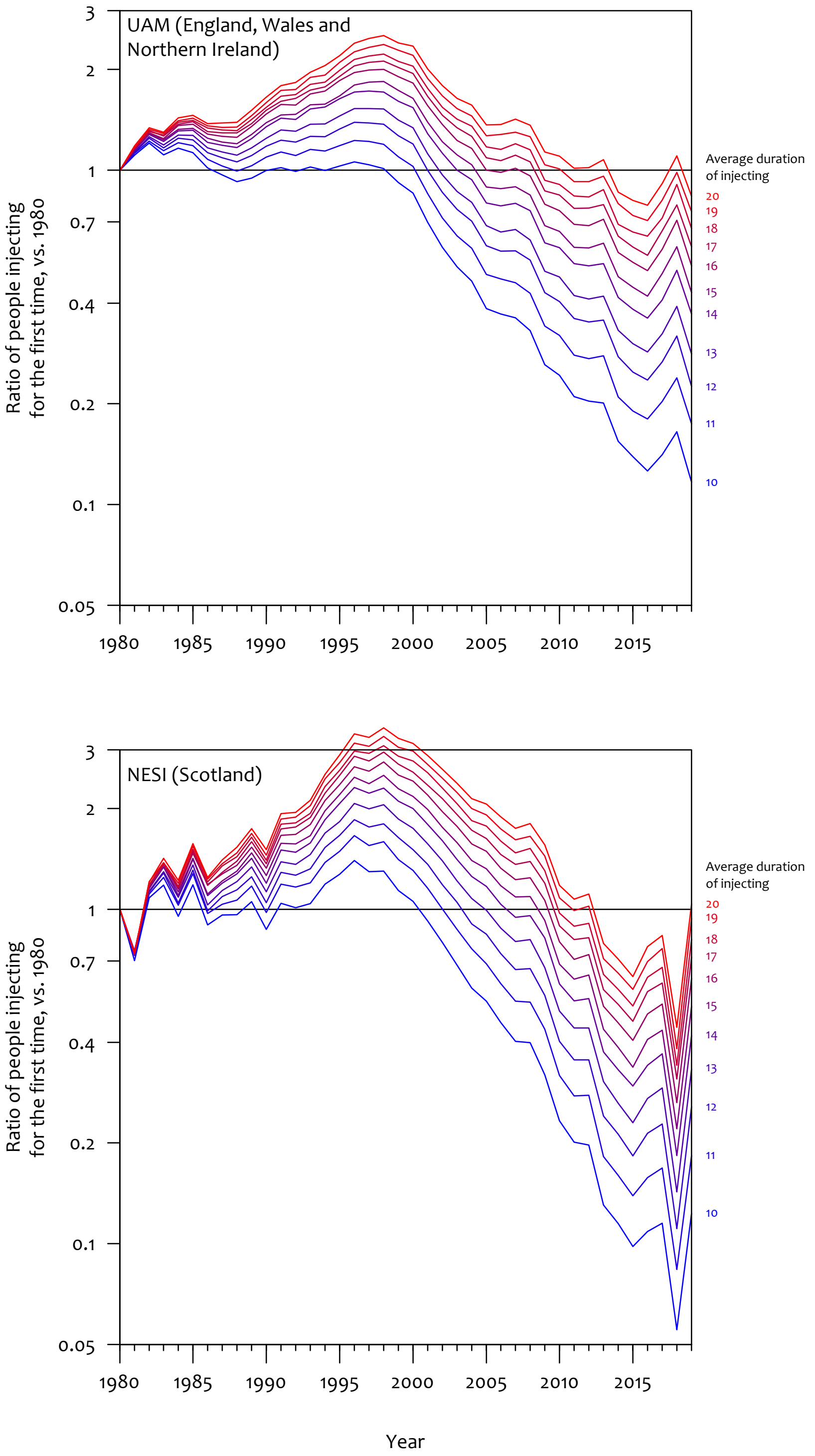

(6) **Use existing estimates of the number of PWID to translate relative cohort sizes into absolute number of people starting injecting.** Previous research has used capture-recapture methods to

estimate that there were 87,300 people injecting illicit drugs in England in 2011 and 23,933 people injecting illicit drugs in Scotland in 2006.[1,2] These estimates also include regional values (Table 5). If we guess the number of people first injecting in 1980, we can use the ratios of new cohort sizes in subsequent years and the assumption that 1/15 stop injecting each year to calculate the population size in each year. We used trial-and-error to set the size of the new cohort in 1980 that produced the population estimate in the relevant year (e.g. 6,650 in 2011 for East of England). We repeated this process for each of the 1000 estimates of the ratio of new cohort sizes. We also incorporated uncertainty in the population estimates by sampling these estimates from a distribution based on the published confidence interval. We estimated each region separately and there are small differences between the estimates for Scotland and England and the total of estimates for regions within each country.

Table 5: Estimates of the number of people who inject drugs, from previous research

Country and year of estimate	Region	Point estimate	95% confidence interval
England (2011)[1]	England	87,302	85,307-90,353
	East of England	6,650	5,995-7,386
	East Midlands	7,808	6,989-8,630
	London	11,351	10,711-12,347
	North East	6,334	5,948-6770
	North West	13,110	12.233-14,305
	South East	11,047	9,635-12,368
	South West	10,134	9,474-10,958
	West Midlands	9,175	8,281-10,082
	Yorkshire & Humber	11,692	11,024-12,457
Scotland (2006)[2]	Scotland	23,933	21,655-27,143
	Lothian & Tayside	4,516	3,536-5,962
	Greater Glasgow & Clyde	8,862	7,091-11,330
	Other	10,354	7,857-10,517

(7) Sensitivity analysis of modelling in England, restricting the survey years contributing data to the model. The modelling in Scotland is based on NESI surveys conducted from 2008 onwards. This does allow estimation of the number of people injecting for the first time from 1980 onwards, because participants report the year when they first injected and/or the duration of injecting. We explored the possible impact of using data from 2008 onwards by comparing the main results for England (which uses UAM surveys from 1990 onwards) with a model for England using data from 2008 only. The results comparing these two scenarios are almost identical in later years (after 2008 the difference is only due to bootstrapping procedures described above), and in earlier years there are minor differences between the main analysis and the results using data from 2008. This may suggest that the relative number of people injecting for the first time in the 1980s and 1990s can be estimated from surveys conducted in the 2000's. Note that NESI runs every two years, while UAM is annual, and therefore the sensitivity analysis using data from 2008-2019 has a slightly different date range to NESI.

Additionally, to explore the impact of using our method with more limited data, we restricted survey data to the most recent year (i.e. 2019), the most recent two years (i.e. 2018-19), the most recent three years, etc. The results in each scenario show similar numbers of people injecting for the first time each year, though models using fewer survey years are more volatile and have wider uncertainty intervals. This may suggest (a) our method can be used where fewer survey years available, and (b) the assumption of constant cessation rate is reasonable (since a varying cessation rate would likely lead to different results in these sensitivity analyses). The results are shown in Figure 8.

Figure 8: Sensitivity analysis restricting the UAM surveys that data to the model of new initiators

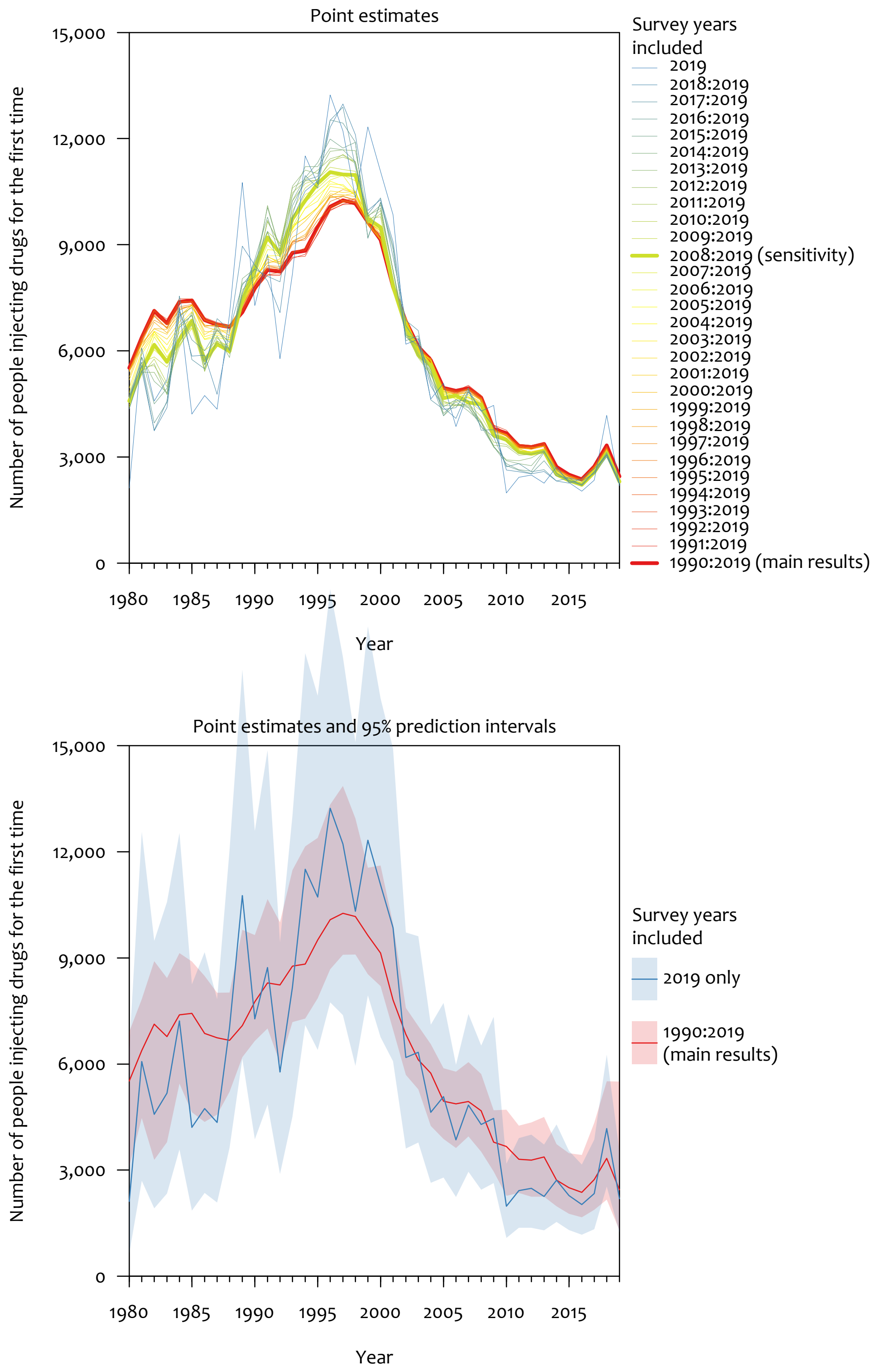

6. Estimated number of people injecting drugs for the first time in England, Scotland, and subregions, 1980-2019

Table 6: Estimated number of people injecting drugs for the first time in England (all regions), East of England, East Midlands, London, and the North East (95% prediction interval)

Year	England	East of England	East Midlands	London	North East
1980	5470 (3120-6940)	520 (250-1040)	380 (190-900)	1690 (950-2690)	200 (110-1000)
1981	6340 (4520-7890)	770 (450-1350)	390 (190-730)	1880 (950-3050)	210 (120-1270)
1982	7060 (3270-8790)	990 (380-1550)	380 (210-670)	1870 (510-2350)	260 (140-670)
1983	6710 (3760-8430)	870 (290-1450)	400 (170-710)	1600 (610-2090)	170 (100-540)
1984	7250 (5530-9040)	780 (440-1190)	350 (210-500)	1660 (980-2960)	210 (110-580)
1985	7320 (4730-8860)	490 (270-760)	390 (230-780)	1780 (1130-2810)	180 (110-530)
1986	6770 (4450-8400)	530 (280-780)	430 (230-660)	1280 (380-1680)	210 (140-520)
1987	6730 (4740-8010)	430 (200-680)	440 (280-740)	1460 (690-2180)	160 (100-420)
1988	6640 (5200-7980)	480 (270-790)	490 (260-930)	1230 (500-1600)	210 (150-440)
1989	7090 (6110-10120)	500 (250-690)	550 (340-920)	1330 (920-2270)	240 (140-1170)
1990	7760 (6590-9590)	520 (270-740)	560 (360-860)	1400 (910-2220)	290 (190-550)
1991	8240 (6950-10710)	630 (370-960)	550 (340-800)	1330 (840-2350)	350 (210-600)
1992	8240 (6150-9720)	650 (340-910)	720 (460-1050)	1320 (710-1840)	390 (230-600)
1993	8770 (7100-11310)	620 (350-860)	810 (520-1360)	1340 (850-2170)	520 (320-800)
1994	8990 (7220-12220)	750 (490-1270)	890 (490-1210)	1160 (860-2120)	560 (370-910)
1995	9480 (7910-12400)	760 (500-1130)	920 (610-1380)	1090 (650-1580)	660 (480-1080)
1996	10060 (8670-13740)	780 (530-1150)	1030 (700-1420)	1050 (610-1490)	730 (440-1080)
1997	10210 (8960-13540)	730 (490-1180)	1020 (700-1430)	1240 (930-2380)	720 (320-1040)
1998	10270 (8980-12780)	710 (460-1000)	970 (620-1310)	1130 (840-1970)	940 (670-1430)
1999	9620 (8520-11520)	610 (350-850)	950 (580-1320)	920 (590-1320)	990 (570-1440)
2000	9220 (8270-11470)	610 (400-960)	1160 (840-1640)	980 (580-1420)	860 (540-1170)
2001	7820 (6970-10040)	480 (330-760)	980 (690-1360)	740 (410-1080)	710 (500-990)
2002	6790 (5630-7570)	520 (320-720)	720 (490-1000)	720 (460-1080)	700 (370-980)
2003	6140 (5260-7050)	450 (300-670)	600 (360-870)	610 (290-850)	610 (410-930)
2004	5790 (4410-6600)	420 (250-650)	500 (300-770)	650 (340-910)	490 (260-690)
2005	4940 (3850-5840)	420 (240-630)	510 (340-780)	450 (160-630)	360 (140-540)
2006	4850 (3730-5790)	370 (220-580)	370 (200-580)	600 (250-860)	350 (200-540)
2007	4970 (3940-6140)	480 (280-710)	410 (250-630)	480 (280-810)	390 (210-580)
2008	4650 (3540-5860)	410 (240-670)	350 (210-530)	590 (310-940)	400 (210-620)
2009	3810 (3010-4820)	320 (170-540)	270 (150-470)	380 (220-730)	250 (140-460)
2010	3660 (2240-4770)	230 (120-430)	260 (150-440)	470 (170-790)	240 (90-410)
2011	3340 (2260-4380)	220 (110-420)	240 (130-410)	300 (170-570)	220 (60-390)
2012	3300 (2310-4450)	300 (160-560)	230 (120-400)	350 (180-750)	210 (70-390)
2013	3380 (2340-4530)	330 (160-700)	220 (110-420)	320 (160-670)	200 (90-440)
2014	2700 (1930-3790)	310 (140-630)	160 (70-340)	250 (120-530)	140 (40-350)
2015	2510 (1790-3480)	230 (110-530)	120 (40-310)	350 (170-810)	230 (70-590)
2016	2410 (1700-3640)	190 (70-460)	90 (40-240)	360 (160-880)	110 (20-370)
2017	2720 (1880-4420)	300 (100-790)	200 (80-560)	310 (120-860)	180 (60-750)
2018	3280 (2170-5840)	350 (70-1150)	150 (30-630)	460 (150-1480)	200 (50-890)
2019	2420 (1320-5580)	190 (40-970)	120 (30-770)	90 (20-520)	260 (50-2040)

Table 7: Estimated number of people injecting drugs for the first time in the North West, South East, South West, West Midlands, and Yorkshire & Humber (95% prediction intervals)

Year	North West	South East	South West	West Midlands	Yorkshire & Humber
1980	1030 (460-2290)	730 (380-1480)	620 (380-2300)	530 (270-850)	750 (370-2000)
1981	1070 (510-1450)	840 (340-1190)	610 (390-1310)	500 (310-770)	710 (390-1240)
1982	1370 (410-1790)	1020 (450-1490)	650 (270-920)	590 (320-940)	820 (470-1350)
1983	1340 (610-1900)	920 (380-1310)	640 (330-1120)	580 (370-900)	620 (320-1390)
1984	1740 (830-2300)	770 (480-1200)	740 (390-1070)	710 (400-1030)	950 (480-1920)
1985	1920 (1280-3330)	980 (380-1460)	740 (470-1260)	550 (340-880)	590 (310-1010)
1986	1640 (810-2610)	700 (240-1000)	720 (430-1170)	540 (240-930)	570 (290-920)
1987	1540 (640-2030)	680 (390-1050)	630 (350-1090)	440 (300-780)	1160 (660-1860)
1988	1620 (680-2040)	580 (360-1260)	690 (440-1280)	570 (360-850)	1300 (750-2030)
1989	1730 (1100-2500)	700 (390-1190)	680 (460-1390)	420 (240-750)	1140 (710-2160)
1990	1760 (1130-2450)	830 (420-1200)	830 (520-1360)	560 (360-850)	1370 (770-2100)
1991	1970 (1320-2820)	890 (590-1720)	820 (550-1750)	740 (470-1040)	1260 (660-1880)
1992	1840 (1240-2620)	780 (170-1090)	930 (600-1470)	450 (280-730)	1220 (460-1750)
1993	1610 (600-2160)	1000 (700-1800)	940 (540-1330)	790 (490-1240)	1550 (950-2200)
1994	1600 (1190-2560)	1160 (720-1790)	1010 (670-1770)	870 (530-1350)	1590 (1030-2320)
1995	1420 (1020-2350)	1400 (830-1920)	1270 (790-1660)	920 (560-1350)	1510 (980-2390)
1996	1440 (1110-2630)	1390 (880-1890)	1360 (950-2020)	950 (610-1410)	1990 (1390-3030)
1997	1380 (1060-2170)	1320 (850-2010)	1410 (980-2010)	1040 (690-1440)	1780 (1090-2600)
1998	1310 (960-1870)	1330 (940-1950)	1350 (980-2150)	1030 (690-1470)	1780 (1250-2620)
1999	1280 (950-2050)	1230 (800-1740)	1010 (500-1350)	1160 (820-1550)	1530 (970-2200)
2000	1060 (750-1580)	1160 (690-1580)	960 (700-1530)	1060 (740-1490)	1360 (900-1970)
2001	1040 (640-1410)	950 (640-1510)	860 (640-1460)	860 (570-1170)	1070 (640-1510)
2002	830 (560-1300)	820 (510-1270)	720 (380-990)	800 (480-1110)	970 (600-1520)
2003	820 (540-1280)	710 (470-1010)	640 (420-900)	820 (550-1160)	640 (380-980)
2004	690 (320-1040)	710 (440-1010)	680 (390-960)	800 (530-1120)	540 (310-810)
2005	600 (300-930)	650 (420-960)	590 (240-810)	600 (380-890)	650 (360-1040)
2006	550 (350-910)	680 (450-1010)	620 (320-900)	570 (370-830)	530 (320-830)
2007	610 (400-1010)	750 (490-1120)	550 (290-810)	610 (410-880)	360 (220-620)
2008	520 (190-830)	630 (360-930)	580 (350-910)	550 (350-810)	370 (190-590)
2009	420 (250-730)	560 (340-850)	480 (170-710)	390 (210-640)	310 (150-510)
2010	410 (210-680)	500 (210-850)	510 (290-820)	460 (270-740)	300 (150-550)
2011	400 (200-690)	460 (260-740)	440 (170-700)	410 (240-640)	290 (140-480)
2012	420 (210-760)	510 (250-860)	340 (130-580)	400 (220-710)	320 (160-580)
2013	440 (220-830)	500 (230-870)	270 (120-550)	420 (220-830)	300 (160-580)
2014	250 (110-500)	440 (230-830)	270 (110-550)	420 (210-890)	200 (100-400)
2015	250 (120-590)	330 (160-710)	250 (90-610)	260 (120-570)	280 (130-610)
2016	350 (160-910)	390 (180-860)	140 (60-350)	330 (130-870)	110 (40-250)
2017	210 (80-660)	280 (100-620)	320 (120-960)	440 (190-1080)	250 (70-760)
2018	500 (170-1760)	520 (220-1630)	240 (80-840)	330 (90-1120)	160 (50-940)
2019	110 (20-840)	510 (150-2300)	200 (40-1300)	330 (80-2050)	260 (50-1200)

Table 8: Estimated number of people injecting drugs for the first time in Scotland (all regions), Greater Glasgow & Clyde, Lothian & Tayside, and other regions of Scotland (95% prediction intervals)

Year	Scotland	Greater Glasgow & Clyde	Lothian and Tayside	Other regions of Scotland
1980	1220 (740-2430)	630 (320-1520)	190 (60-420)	330 (150-1530)
1981	900 (510-1110)	500 (230-690)	140 (70-280)	280 (110-450)
1982	1410 (910-1790)	840 (440-1120)	180 (80-400)	340 (170-520)
1983	1630 (930-1910)	940 (530-1240)	340 (150-620)	490 (190-740)
1984	1380 (700-1610)	900 (480-1160)	200 (90-370)	270 (120-390)
1985	1770 (1120-2000)	990 (500-1240)	200 (90-390)	440 (210-610)
1986	1370 (930-2160)	730 (390-1070)	180 (90-350)	390 (200-620)
1987	1520 (1010-1790)	720 (380-920)	200 (90-460)	500 (260-690)
1988	1620 (950-1830)	790 (420-1030)	220 (80-380)	530 (250-720)
1989	1820 (1040-2040)	950 (470-1170)	260 (110-470)	600 (320-770)
1990	1550 (1040-1760)	730 (400-930)	260 (130-460)	590 (300-780)
1991	1940 (1350-2160)	840 (470-1030)	330 (170-520)	750 (430-930)
1992	1930 (1390-2320)	830 (490-1140)	300 (160-540)	780 (410-1040)
1993	2050 (1470-2290)	950 (550-1150)	230 (110-390)	850 (490-1040)
1994	2420 (1730-2640)	1030 (610-1250)	330 (170-510)	1060 (630-1300)
1995	2700 (1980-3030)	1080 (660-1260)	340 (190-540)	1280 (800-1540)
1996	3040 (2200-3370)	1240 (750-1450)	390 (230-600)	1440 (910-1670)
1997	2970 (2120-3200)	1130 (620-1290)	320 (180-490)	1490 (960-1810)
1998	3080 (2160-3350)	1220 (720-1420)	410 (230-630)	1490 (840-1690)
1999	2800 (1990-3040)	1060 (580-1240)	400 (230-580)	1360 (870-1600)
2000	2680 (1950-2890)	1040 (610-1240)	420 (260-670)	1210 (740-1400)
2001	2440 (1750-2630)	840 (500-1000)	460 (270-690)	1140 (720-1340)
2002	2160 (1600-2410)	640 (380-750)	480 (290-720)	1070 (680-1280)
2003	1950 (1390-2110)	600 (350-710)	440 (240-620)	890 (550-1080)
2004	1730 (1150-1810)	500 (270-590)	430 (250-640)	820 (490-960)
2005	1630 (1150-1780)	460 (260-560)	420 (220-630)	720 (460-920)
2006	1460 (1040-1580)	400 (240-490)	410 (230-580)	630 (390-800)
2007	1330 (940-1470)	380 (230-510)	370 (200-580)	590 (330-680)
2008	1350 (860-1460)	450 (240-530)	360 (200-540)	530 (320-630)
2009	1140 (760-1250)	380 (200-470)	310 (160-460)	430 (240-530)
2010	840 (600-960)	240 (140-330)	230 (110-350)	380 (220-490)
2011	760 (510-900)	240 (130-320)	200 (100-350)	300 (150-410)
2012	780 (480-910)	230 (110-300)	200 (100-490)	350 (180-630)
2013	540 (350-670)	160 (70-230)	150 (60-260)	250 (130-420)
2014	480 (290-650)	130 (60-200)	180 (80-360)	150 (70-300)
2015	420 (230-570)	140 (60-220)	110 (40-240)	220 (80-400)
2016	490 (200-770)	160 (60-260)	190 (50-680)	210 (80-490)
2017	540 (270-850)	140 (50-270)	190 (50-610)	270 (120-710)
2018	270 (130-600)	120 (40-340)	10 (0-50)	120 (40-300)
2019	640 (270-1480)	140 (50-400)	-	460 (150-1330)

7. Histograms of duration of injecting by survey year in Scotland

Figure 9: Duration of injecting illicit drugs among people who inject drugs in Scotland, 2008-2019

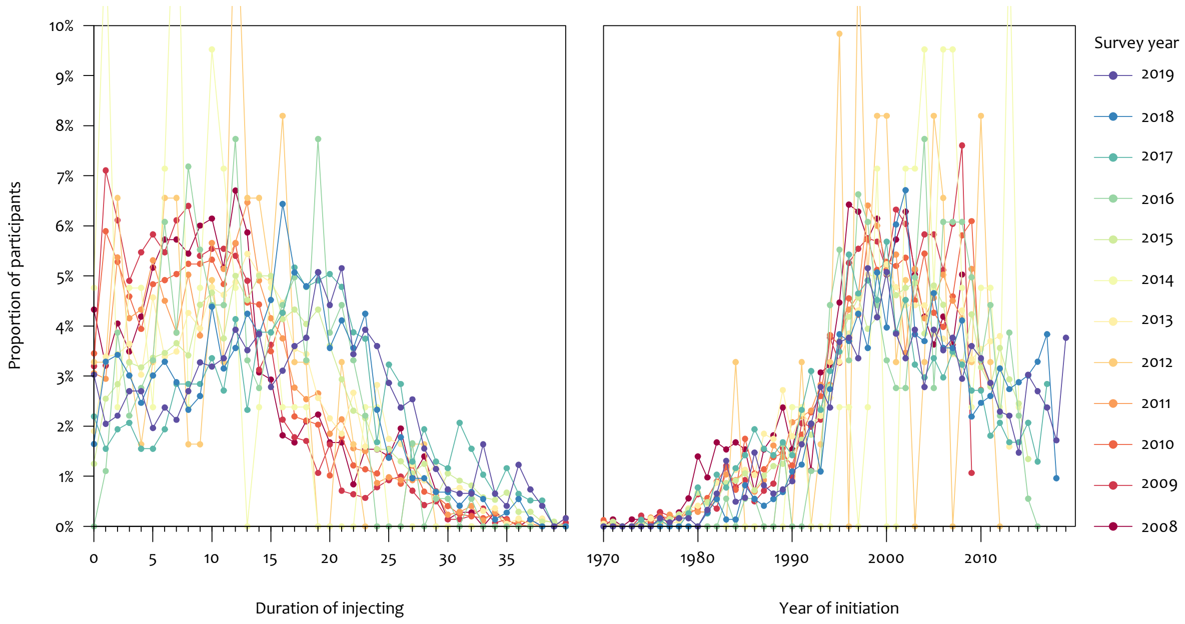

8. References for supplementary information

1 Hay G, Rael dos Santos A, Worsley J. Estimates of the Prevalence of Opiate Use and/or Crack Cocaine Use, 2011/12: Sweep 8 report. 2014.https://core.ac.uk/reader/34721711 (accessed 21 Jun 2019).

2 Hay G, Gannon M, Casey J, *et al.* Estimating the National and Local Prevalence of Problem Drug Misuse in Scotland. 2009.http://eprints.gla.ac.uk/45433/3/45433.pdf
